# Forensic data on adolescents who died by suicide in Greece

**DOI:** 10.1192/j.eurpsy.2021.1550

**Published:** 2021-08-13

**Authors:** A. Paraschakis, V. Karageorgiou, I. Kourtesis, I. Boyokas, I. Michopoulos

**Affiliations:** 1 Department Of General Adult Psychiatry, Psychiatric Hospital of Attica “Dafni”, Athens, Greece; 2 2nd Department Of Psychiatry, “attikon” General Hospital, National and Kapodistrian University of Athens, Athens, Greece; 3 Piraeus Department Of Forensic Medicine, Ministry of Justice, Piraeus, Greece

**Keywords:** Forensic, Suicide, adolescent, Greece

## Abstract

**Introduction:**

Suicide is the second most common cause of adolescent mortality worldwide.

**Objectives:**

To study the characteristics of a sample of adolescents (<18years of age) who died by suicide in Greece.

**Methods:**

We investigated all suicides that took place within the area of the Piraeus Department of Forensic Medicine (population covered ~700,000) for the period 1992-2016, based on the victims’ forensic records.

**Results:**

During the 25-year period, 16 adolescents (and 1162 adults) died by suicide. They were mostly males (11/68.75%) and of Greek Nationality (14/87.5%). The mean age was 15.56 years (range: 12-17, standard deviation: 1.46). Two (12.5%) were under psychiatric medication (an antipsychotic and an antidepressant, respectively); none was receiving a benzodiazepine or a mood-stabilizing antiepileptic. None had used amphetamine, cannabis, cocaine or heroin. Two (12.5%) -one girl one boy- had consumed alcohol. The suicides took place primarily at home (12/75%), followed by outdoors (3/18.75%); one (6.25%) took place in a correctional facility. Hanging was the most prevalent method (6/37.5), followed by jumping (5/31.25%), shooting by a firearm (2/12.5%), drowning (1/6.25%), medication overdose (1/6.25% -amitriptyline poisoning) and a case of suffocating death (1/6.25%). Most suicides happened in September (5/31.25%) and April (3/18.75%). No significant differences were noted with the adult sample.

**Conclusions:**

The methods chosen by the adolescents who died by suicide in our sample differ strikingly from those of usual suicide attempts at that age (medication overdose/self-cutting). The periods when the suicides took place may imply a role for school stress. Our study was retrospective and focused primarily on a large urban area.

